# Adverse Effects Associated With the Use of Antimalarials During The COVID-19 Pandemic in a Tertiary Care Center in Mexico City

**DOI:** 10.3389/fphar.2021.668678

**Published:** 2021-06-03

**Authors:** Oscar Arturo Lozano-Cruz, José Víctor Jiménez, Antonio Olivas-Martinez, Edgar Ortiz-Brizuela, José Luis Cárdenas-Fragoso, Daniel Azamar-Llamas, Sergio Rodríguez-Rodríguez, Jorge Carlos Oseguera-Moguel, Joel Dorantes-García, Clemente Barrón-Magdaleno, Aldo C Cázares-Diazleal, Carla Marina Román-Montes, Karla María Tamez-Torres, Bernardo Alfonso Martínez-Guerra, Alfonso Gulias-Herrero, María Fernanda González-Lara, Alfredo Ponce-de-León-Garduño, David Kershenobich-Stalnikowitz, José Sifuentes-Osornio

**Affiliations:** ^1^ Department of Medicine, Instituto Nacional de Ciencias Médicas y Nutrición Salvador Zubirán, Mexico City, Mexico; ^2^ Department of Biostatistics, University of WA, Seattle, WA, United States; ^3^ Department of Cardiology, Instituto Nacional de Ciencias Médicas y Nutrición Salvador Zubirán, Mexico City, Mexico; ^4^ Department of Infectious Diseases, Instituto Nacional de Ciencias Médicas y Nutrición Salvador Zubirán, Mexico City, Mexico; ^5^ General Director’s Office, Instituto Nacional de Ciencias Médicas y Nutrición Salvador Zubirán, Mexico City, Mexico

**Keywords:** hydroxychloroquine, chloroquine, COVID-19, arrythmia, adverse-effects, antimalarial

## Abstract

**Background:** Antimalarial drugs were widely used as experimental therapies against COVID-19 in the initial stages of the pandemic. Despite multiple randomized controlled trials demonstrating unfavorable outcomes in both efficacy and adverse effects, antimalarial drugs are still prescribed in developing countries, especially in those experiencing recurrent COVID-19 crises (India and Brazil). Therefore, real-life experience and pharmacovigilance studies describing the use and side effects of antimalarials for COVID-19 in developing countries are still relevant.

**Objective:** To describe the adverse effects associated with the use of antimalarial drugs in hospitalized patients with COVID-19 pneumonia at a reference center in Mexico City.

**Methods:** We integrated a retrospective cohort with all adult patients hospitalized for COVID-19 pneumonia from March 13th, 2020, to May 17th, 2020. We compared the baseline characteristics (demographic and clinical) and the adverse effects between the groups of patients treated with and without antimalarial drugs. The mortality analysis was performed in 491 patients who received optimal care and were not transferred to other institutions (210 from the antimalarial group and 281 from the other group).

**Results:** We included 626 patients from whom 38% (*n* = 235) received an antimalarial drug. The mean age was 51.2 ± 13.6 years, and 64% were males. At baseline, compared with the group treated with antimalarials, the group that did not receive antimalarials had more dyspnea (82 vs. 73%, *p* = 0.017) and cyanosis (5.3 vs. 0.9%, *p* = 0.009), higher respiratory rate (median of 28 vs. 24 bpm, *p* < 0.001), and lower oxygen saturation (median of 83 vs. 87%, *p* < 0.001). In the group treated with antimalarials, 120 patients had two EKG evaluations, from whom 12% (*n* = 16) prolonged their QTc from baseline in more than 50 ms, and six developed a ventricular arrhythmia. Regarding the trajectories of the liver function tests over time, no significant differences were found for the change in the mean value per day between the two groups. Among patients who received optimal care, the mortality was 16% (33/210) in those treated with antimalarials and 15% (41/281) in those not receiving antimalarials (RR 1.08, 95% 0.75–1.64, and adjusted RR 1.12, 95% CI 0.69–1.82).

**Conclusion:** The adverse events in patients with COVID-19 treated with antimalarials were similar to those who did not receive antimalarials at institutions with rigorous pharmacological surveillance. However, they do not improve survival in patients who receive optimal medical care.

## Introduction

Hydroxychloroquine (HCQ) and chloroquine (CLQ) are antimalarial drugs recently repurposed as a possible therapy against COVID-19 due to their immunomodulatory properties and the *in-vitro* antiviral effect against SARS-CoV-2 observed in experimental models (Chowdhury et al., 2020; Gautret et al., 2020). As a consequence, the Food and Drug Administration (FDA) provided an emergency authorization use against SARS-CoV-2 infection (US Food and Drug Administration, 2021a), which was later revoked (US Food and Drug Administration, 2021b) due to the negative results observed in randomized controlled trials (RCTs) (Boulware et al., 2020; Mehra et al., 2020a). The World Health Organization (WHO) withdrew hydroxychloroquine from its clinical trial *Solidarity* in July 2020 (RECOVERY Collaborative Group et al., 2020; Borba et al., 2021; Geleris et al., 2020; Self et al., 2020). In addition, several trials have demonstrated a high prevalence of significant adverse effects (primarily cardiovascular) in patients receiving this drug (RECOVERY Collaborative Group et al., 2020; WHO.int, 2021). Despite the compelling evidence, antimalarial drugs have resurged in developing countries experiencing recurrent outbreaks. On the past April 22, 2021, the AIIMS/ICMR—COVID 19 National Task Force/Joint Monitoring Group of the Ministry of Health and Family Welfare of India updated the clinical guidelines for managing adult patients with COVID-19 considering both ivermectin and HCQ in the category of “May Do” with low certainty of evidence (Icmrgovin, 2021). Similarly, the Brazilian government provides a “Covid-Kit” consisting of antimalarials and ivermectin (Kmietowicz, 2021).

Overall, antimalarial drugs are safe. The most frequent adverse effects are nausea, diarrhea, headache, diplopia, pruritus, urticaria, lichenoid rash, hair discoloration, seizures, and anxiety. The accumulation of high doses ( >1 g/kg), usually due to a prolonged use, may develop ototoxicity, retinopathy, myopathy, heart toxicity, and peripheral neuropathy (Mercuro et al., 2020; Tang et al., 2020). Less frequently, acute and potentially fatal adverse effects such as QT prolongation, T wave abnormalities, and vasodilation may occur (Rosenberg et al., 2020; Tang et al., 2020). The current FDA-approved indications for these medications are predominantly in the ambulatory setting (both as an immunomodulatory medication for autoimmune disorders or as malaria prophylaxis) and rarely in the clinical context of hospitalized patients.

We aimed to describe the prevalence and severity of adverse effects in a cohort of patients with severe COVID-19 who received antimalarial drugs as therapy in a tertiary care center in Mexico City.

## Materials and Methods

### Treatment and Study Design

We retrospectively collected data regarding treatment and adverse effects, as well as baseline characteristics, complications and mortality, from the institutional COVID-19 cohort (Ortiz-Brizuela et al., 2020), which included hospitalized patients from March 13th, 2020 to May 17th, 2020. Antimalarial drug administration was allowed by institutional protocol during the study period and was prescribed by the treating medical team in agreement with patients after a discussion regarding potential risks and benefits. Due to the shortage of HCQ, the most used antimalarial drug was CLQ. The initial dose was 400 mg bid the first day, then 200 mg bd for 5–14 days for HCQ and 300 mg bid on day 1, and then 150 mg bd for CLQ, or until an adverse effect appeared.

This study was approved by the Institutional Review Board (Comité de Investigación and Comité de Ética en Investigación, reference number 3333), who waived the informed consent requirement due to the minimal risk characteristics of an observational study. All the patients admitted to our institution during the pandemic agree with releasing their medical data (*via* standardized consent) for research purposes (and had the option to decline).

### Patients and Follow up

During the hospital stay, patients were clinically evaluated twice a day, at least. Patients had blood testing on days 3 and 7 to assess clinical status and toxicity [complete blood count, blood glucose, serum ferritin, creatine phosphokinase, D-dimer, kidney and liver function tests (LFT), as well as prothrombin time and partial thromboplastin time]. The adverse effects regarding laboratorial testing were classified according to the FDA score: grade 1 (mild), grade 2 (moderate), grade 3 (severe) and grade 4 (life threatening) (US Food and Drug Administration, 2021c), and the specific definitions were: hypoglycemia G1 (55 mg/dl), G2 (<55–40 mg/dl), G3 (<40–30 mg/dl) and G4(<30 mg/dl); neutropenia G1 (2,000–1,500/ mm³), G2 (1,500–1,000/ mm³), G3 (1,000–500/ mm³), G4 (<500/ mm³); leukopenia G1 (4,000–3,000 mm³), G2 (<3,000–2,000/ mm³, G3(<2,000–1,000/ mm³, G4 (<1,000/ mm³); lymphopenia G1 (1,000–800/ mm³, G2 (<800–500/ mm³), G3 (<500–200/ mm³), G4 (<200 mm³); and thrombocytopenia G1 (150,000–75,000/ mm³), G2 (<75,000–50,000/ mm³), G3 (<50,000–25,000/ mm³) and G4 (<25,000/ mm³).

We performed active surveillance of pre-existing arrhythmias through an initial electrocardiogram (EKG) recorded before the first dose for most of the patients and after the second dose for those with borderline QTc in the baseline EKG. Patients were monitored *via* telemetry, abnormal tracing noted by the treating physician triggered additional EKG recordings for further analysis. The medication was stopped in case of ventricular polymorphism. We intentionally looked for ventricular arrhythmias and considered as positive the presence of ventricular premature activity and monomorphic ventricular tachycardia (Zipes et al., 2019). The information related to adverse effects was obtained retrospectively from the electronic records and from laboratory databases. The EKGs were analyzed by experienced cardiologists (JOM, CBM, JDG, ACD).

### Statistical Analysis

Numerical variables are described in mean and standard deviation or in median and interquartile range (IQR) as appropriate, categorical variables are described in frequencies and percentages. The clinical and demographic characteristics on admission were compared between groups defined by the treatment received (antimalarial vs. no antimalarial) through Student’s *t* test, Mann Whitney test or chi square test as appropriate. To identify potential hepatoxicity due to the use of antimalarial, we compare the dynamic profile in LFT between groups defined by the treatment received using generalized linear mixed models adjusted for age, sex, baseline laboratory values and allowing for interaction between time and therapy received (antimalarial vs. no antimalarial). This analysis was performed only on patients with at least two liver function test measurements and we assumed the missing data as missing at random. The mortality analysis was performed in patients who received optimal medical care and who either died or were discharged to home. That is, we excluded patients with ICU requirements who were not admitted to the ICU (due to not intubate/resuscitate order or to lack of ICU-bed) as well as those who were discharged against medical advice or transferred to another institution. The effect of the therapy received (antimalarial vs. no antimalarial) was estimated using Targeted Maximum Likelihood Estimation with a super learning algorithm for the treatment assignment model, adjusting for age, sex, diabetes, body mass index (BMI), period time of admission (before April 15th vs. April 15th or after) and NEWS score (this includes respiratory rate, oxygen saturation, need for supplemental oxygen, heart rate, systolic blood pressure, and responsiveness) and site of admission as confounders. A two-sided *p* value of less than 0.05 was considered statistically significant. All the analyses were performed using R software, version 3.6.3.

## Results

### Demographic Characteristics

During the study period, 626 patients were hospitalized, of whom 235 (37.5%) received an antimalarial drug. Table 1 shows the baseline characteristics, mean age was 51.2 ± 13.6 years and 64% were male. Overall, the most frequent comorbidities were obesity in 48%, overweight in 38%, hypertension in 31% and diabetes in 26%. When comparing baseline characteristics between both groups, only the BMI was significantly different, being lower in patients taking antimalarial drugs (mean of 30 vs. 31 kg/m^2^, *p* = 0.023).

**TABLE 1:** Demographic characteristics and comorbidities of patients with COVID-19 treated with and without antimalarial drugs in a tertiary care center in Mexico City.

Characteristics	*N*	Overall (*N* = 626)	HCQ/CLQ (*N* = 235)	No antimalarial (*N* = 391)	*p*–value
S	626	51.2 ± 13.6	50.0 ± 13.2	52.0 ± 13.8	0.062
Male gender, no.—(%)	626	402 (64)	152 (65)	250 (64)	0.92
BMI, mean (SD)—kg/m^2^	591	30.6 ± 5.8	29.9 ± 5.6	31.0 ± 5.8	0.023
Diabetes—no. (%)	626	166 (26)	59 (25)	107 (27)	0.60
Hypertension—no. (%)	626	194 (31)	68 (28)	126 (32)	0.44
COPD—no. (%)	626	5 (0.8)	2 (0.9)	3 (0.8)	>0.99
CVD—no. (%)	625	26 (4.2)	10 (4.3)	16 (4.1)	>0.99
CKD—no. (%)	626	16 (2.6)	6 (2.6)	10 (2.6)	>0.99
Immunosuppression—no. (%)	626	29 (4.6)	12 (5.1)	17 (4.3)	0.81
Smoking—no. (%)	621	105 (16.9)	44 (18.9)	61 (15.7)	0.36

HCQ, hydroxychloroquine; CLQ, chloroquine; BMI, body mass index; COPD, chronic obstructive pulmonary disease; CVD, cardiovascular disease; CKD, chronic kidney disease; HIV, human immunodeficiency virus.

### Clinical Manifestations and Laboratory Findings

The clinical and laboratorial findings at admission are summarized in Tables 2, 3, respectively. Patients not receiving antimalarials had more cyanosis (5.3 vs. 0.9%, *p* = 0.009), higher respiratory rate (median of 28 vs. 24 bpm, *p* < 0.001) lower oxygen saturation (median of 83 vs. 87%, *p* = 0.001) and higher NEWS score (mean of 8.7 vs. 7.6, *p* < 0.001). Regarding the laboratory findings, patients not receiving antimalarials had higher leukocyte counts (*p* = 0.004), absolute neutrophils count (*p* < 0.001), platelets count (*p* = 0.001), DHL (*p* < 0.001), C-reactive protein (*p* < 0.001), procalcitonin (*p* = 0.003), ferritin (*p* = 0.004), fibrinogen (*p* < 0.001), D-Dimer (D-D) (*p* < 0.001), troponin (*p* < 0.001), lactate serum concentration (*p* < 0.001) and lower PaO2/FiO2 index (*p* < 0.001). Patients in both groups received empiric antibiotics (including macrolides), corticosteroids, anticoagulant therapy, and were enrolled in clinical trials for COVID-19 therapies in the same proportion; however, more patients in the group of antimalarial drugs received oseltamivir (Supplementary Table S1).

**TABLE 2:** Clinical manifestations, physical findings and value of the severity scales on admission on admission of patients with COVID-19 treated with and without antimalarial drugs in a tertiary care center in Mexico City.

Characteristic	*N*	Overall (*N* = 626)	HCQ/CLQ (*N* = 235)	No antimalarial (*N* = 391)	*p*-value
Symptoms—no. (%)					
Fever	625	548 (87.7)	212 (90.2)	336 (86.2)	0.17
Cough	623	569 (91.3)	220 (93.6)	349 (89)	0.15
Headache	620	471 (76)	177 (76)	294 (76)	>0.99
Dyspnea	624	492 (78)	173 (73)	319 (82)	**0.017**
Chest pain	611	206 (33)	75 (32)	131 (34.6)	0.63
Cyanosis	608	22 (3.6)	2 (0.9)	20 (5.3)	**0.009**
Physical findings					
Temperature—mean (SD)—°C	612	37.2 ± 0.8	37.2 ± 0.8	37.2 ± 0.8	0.36
Heart rate—mean—(SD)—bpm	624	102 ± 17.5	101.4 ± 17.5	102.6 ± 17.5	0.40
Respiratory rate, median (IQR)—bpm	623	27 (22–32)	24 (20–30)	28 (24–35)	**<0.001**
Mean arterial pressure, mean (SD)—mmHg	614	91 ± 11.7	90 ± 11.6	91 ± 11.8	0.68
Oxygen saturation, median (IQR)—%	602	85 (74.0–88)	87 (81–89)	83 (70–88)	**<0.001**
Time from symptoms to admission, median (IQR)—days	626	7 (5–10)	7 (5–9)	7 (6–10)	0.075
qSOFA	618				**<0.001**
0		110 (18%)	63 (27%)	47 (12%)	
1		455 (74%)	154 (66%)	301 (78%)	
2		50 (8.1%)	17 (7.3%)	33 (8.6%)	
3		3 (0.5%)	0 (0%)	3 (0.8%)	
Severity scales on admission					
NEWS	617	8.27 (2.28)	7.55 (2.48)	8.72 (2.03)	**<0.001**
NIH Severity	623				**0.006**
Moderate		24 (3.9%)	16 (6.8%)	8 (2.1%)	
Severe		573 (92%)	206 (88%)	367 (94%)	
Critical		26 (4.2%)	12 (5.1%)	14 (3.6%)	

SD, standard deviation; IQR, interquartile range HCQ, hydroxychloroquine; CLQ, chloroquine; qSOFA, quick sequential organ failure assessment; NEWS, National Early Warning Score. Bold values are the statistically significant variables.

**TABLE 3:** Laboratory findings on admission of patients with COVID-19 treated with and without antimalarial drugs in a tertiary care center in Mexico City.

Characteristic	*N*	Overall (*N* = 497)	HCQ/CLQ (*N* = 211)	No antimalarial (*N* = 286)	*p*-value
Hemoglobin, g/dl	617	15.3 ± 2.0	15.4 ± 1.9	15.3 ± 2.0	0.41
Leukocytes, × 10^3^/µl	615	7.9 (5.8–11.0)	7.1 (5.3–9.9)	8.2 (6.1–12.4)	**0.001**
Absolute neutrophil count	613	6,438 (4,468–9,523)	5,833 (4,026–8,483)	6,757 (4,802–10,545)	**<0.001**
Absolute lymphocyte count	613	800 (558–1,057)	806 (560–1,078)	799 (558–1,041)	0.094
Platelets, K/µl	615	214 (174–275)	204 (165–256)	226 (179–292)	**0.001**
BUN, mg/dl	617	15.4 (11.1–22.8)	14.3, (10.6–20.1)	16.0 (11.6–23.9)	**0.006**
Creatinine, mg/dl	617	0.9 (0.8–1.2)	0.9 (0.8–1.2)	1.0 (0.8–1.2)	0.53
Total bilirubin, mg/dl	608	0.7 ± 0.5	0.7 ± 0.5	0.7 ± 0.5	0.31
Albumin, g/dl	607	3.7 ± 0.5	3.8 ± 0.5	3.6 ± 0.5	**0.004**
Globulin, g/dl	604	3.3 ± 0.5	3.2 ± 0.5	3.3 ± 0.5	**0.002**
ALT, u/l	608	36 (24–55)	34 (23–53)	38 (25–58)	0.11
AST, u/l	608	43 (30–64)	41 (27–60)	44 (31–66)	**0.026**
ALP, u/l	608	88 (70–114)	82 (67–106)	92 (74–118)	**<0.001**
LDH, u/l	573	382 (290–504)	348, (261–479)	395, (313–537)	**<0.001**
CRP, mg/dl	596	14 (7–22)	13 (5–20)	15 (8–23)	**<0.001**
CPK, u/l	538	116 (63–239)	112 (61–238)	116 (64–245)	0.79
Ferritin, ng/ml	588	629 (320–1,066)	539 (250–910)	704 (350–1,105)	**0.004**
Fibrinogen, mg/dl	508	688 (499–834)	618 (468–774)	715 (554–883)	<**0.001**
D-dimer, ng/ml	584	701 (437–1,138)	568 (390–1,020)	770 (595–1,208)	**<0.001**
Troponin I, pg/ml	532	5.5 (3.6–11.4)	5.0 (3.5–7.3)	6.1 (3.8–13.6)	**<0.001**
pO2, mmHg	599	62.9 (54.0–77.8)	63.8 (55.2–76.0)	62.5 (52.8–76.0)	0.077
Lactate, mmol/L	470	1.3, (1.0–1.9)	1.2, (0.9–1.6)	1.5, (1.1, 2.1)	**<0.001**
PaO2/FiO2 index	595	209 (124–266)	235 (160–281)	185 (109–250)	**<0.001**

HCQ, hydroxychloroquine; CLQ, chloroquine; BUN, blood urea nitrogen; ALT, alanine transaminase; AST aspartate transaminase; ALP, alkaline phosphatase; LDH, lactate dehydrogenase; CRP, C reactive protein; CPK, creatine phosphokinase; pO2, partial pressure of oxygen; FiO2, fraction of inspired oxygen. Bold values are the statistically significant variables.

#### Adverse Effects

During the hospitalization period, there were no significant differences between both groups in the presence of hypoglycemia; however, two patients receiving antimalarials had grade-4 hypoglycemia. Although there were not significant differences in cytopenias between both groups, severe cases of lymphopenia and neutropenia were more frequent in the group receiving antimalarial drugs and only one case of grade-3 thrombocytopenia was observed in both groups (Table 4). No neurological effects were reported.

**TABLE 4:** Adverse effects of patients with COVID-19 treated with and without antimalarial drugs in a tertiary care center in Mexico City.

	*N*	Overall, *N* = 626	HCQ/CLQ, *N* = 235	No antimalarial, *N* = 391	*p*-value
Hypoglycemia, grade	494				0.57
0		473 (96%)	200 (95%)	273 (96%)	
1		10 (2.0)	5 (2.4%)	5 (1.8%)	
2		4 (0.8%)	1 (0.5%)	3 (1.1%)	
3		5 (1.0%)	2 (1.0%)	3 (1.1%)	
4		2 (0.4%)	2 (1.0%)	0 (0%)	
Leukopenia, grade	453				0.074
0		404 (89%)	171 (86%)	233 (92%)	
1		38 (8.4%)	23 (12%)	15 (5.9%)	
2		11 (2.4%)	6 (3.0%)	5 (2.0%)	
3		0	0	0	
4		0	0	0	
Neutropenia, grade	451				0.41
0		422 (94%)	185 (92%)	237 (94%)	
1		22 (4.9%)	10 (5.0%)	12 (4.8%)	
2		7 (1.6%)	5 (2.5%)	2 (0.8%)	
3		0	0	0	
4		0	0	0	
Thrombocytopenia, grade	453				0.90
0		409 (90%)	179 (90%)	230 (91%)	
1		41 (9.1%)	20 (10%)	21 (8.3%)	
2		1 (0.2%)	0 (0%)	1 (0.4%)	
3		2 (0.4%)	1 (0.5)	1 (0.4%)	
4		0 (0%)	0 (0%)	0 (0%)	

HCQ, hydroxychloroquine; CLQ, chloroquine.

#### EKG Alterations

A baseline EKG was obtained in 292 patients (177 from the antimalarial group and 115 from the group without antimalarials) from whom 132 had a follow-up EKG (120 in the group receiving antimalarials and 12 in the group without antimalarials). In the group receiving antimalarials, 13% (16/120) prolonged their QTc at least 50 ms and six developed a ventricular arrhythmia. From the 12 patients with a follow-up EKG in the group not receiving antimalarials, no one prolonged their QTc in more than 50 ms and two developed a ventricular arrhythmia. The characteristics of the eight patients in whom serious arrhythmias were documented are described in Supplementary Table S3.

#### Dynamic Profile of Liver Function Tests

**FIGURE 1:**
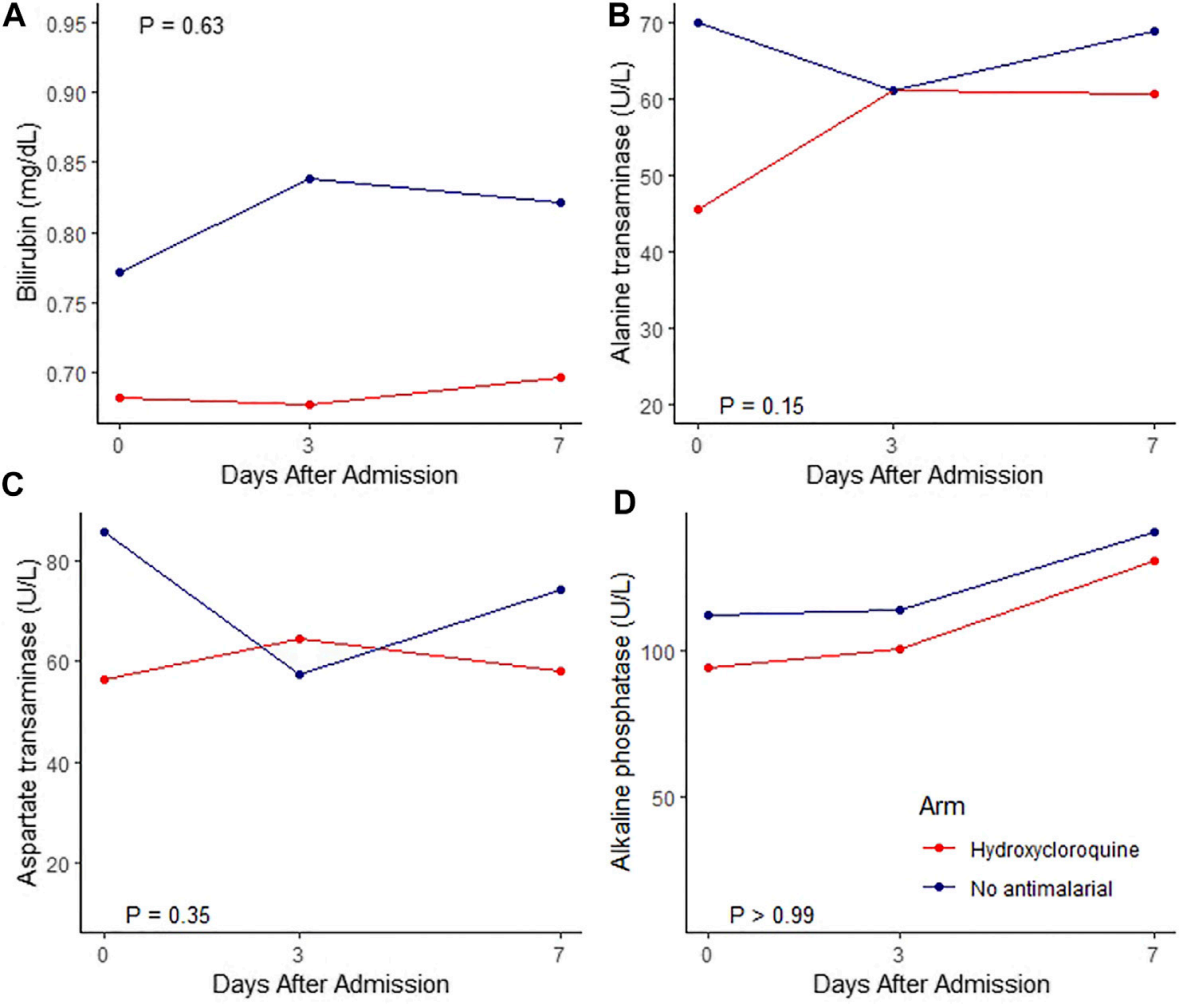
Linear Mixed Effect Modelor for bilirubin, alanine transaminase, aspartate transaminse aminotransferase, alkaline phosphatase adjusted for age, sex and basal. Red line: hydroxycloroquine, Blue line: no antimalarial.

During their follow-up, 308 patients had at least two LFT determinations, 132 (43%) from the HCQ/CLQ group and 176 (57%) from the group not receiving antimalarials. The dynamic profiles of the total bilirubin, alanine transferase (ALT), aspartate transferase (AST) and alkaline phosphatase (ALP) are displayed in Figure 1; the change in the mean value per day for each of these LFT was not significantly different between groups.

#### Effect on Mortality

The mortality analysis was performed on 491 patients who received optimal care, 210 from the HCQ/CLQ group and 281 from the other group. The risk of in-hospital mortality was 16% (33/210) in patients who received HCQ/CLQ and 15% (41/281) in those who did not, with a risk ratio of 1.08, 95% CI 0.71—1.64, and an adjusted risk ratio of 1.12, 95% CI 0.69—1.82. See Supplementary Table S4 for futher information.

## Discussion

This is the first real-life experience and pharmacovigilance study describing the use and side effects of antimalarials for COVID-19 in Mexico and Latin America during the early months of the pandemic. Despite patients not receiving antimalarials were more severely ill than those receiving antimalarials; the adverse effects regarding hypoglycemia, liver function tests and cytopenias were similar between both groups. Furthermore, when restricting to patients that received optimal care, both groups had similar mortality. This provides evidence against the use of antimalarials for COVID-19; they do not improve mortality over optimal care and their adverse effects are comparable with those experienced by more severely ill patients.

The disparity in disease severity at admission between both groups might be explained by the fact that the therapy was decided by the treating physician. It is possible that those patients that the treating physicians observed less severe were proposed treatment with antimalarials, while the more serious patients were avoided the risk of any potential arrhythmia with the use of the antimalarial.

Although antimalarials have shown an acceptable safety profile in the treatment of malaria, recent studies in COVID-19 have reported significant cardiac effects (Haeusler et al., 2018; Chowdhury et al., 2020). The most reported cardiac effect is prolongation of the QT interval, that increases the risk of torsade de pointes or sudden death (Patil et al., 2020), and it can be triggered by the concomitant use of macrolides as occurred in the early months of the pandemic. (Mason, 2017; Jeevaratnam, 2020). In a study performed in New York City, Mehra et al. reported that 23% of the patients who received antimalarials plus azithromycin prolonged their QTc interval in at least 60 ms (Mehra et al., 2020b). In the same way, in another study performed in New York City, Chorin et al. reported that, out of 84 patients who received antimalarials plus azithromycin, 30% prolonged their QTC more than 40 ms and 11% prolonged their QTc more than 50 ms (Chorin et al., 2020). In our study, 13% of patients receiving antimalarials prolonged their QTc in more than 50 ms; however, no additional electrocardiographic follow-up was performed. Furthermore, we detected eight ventricular arrhythmias, all in the context of hypoxemia and concomitant administration of macrolides, six occurred in the group receiving antimalarials from whom three died, and two in the group not receiving antimalarials from whom both died (Supplementary Table S2). Although it seems ventricular arrhythmias were more fatal in patients not receiving antimalarials, the follow-up EKG in these patients was recorded due to clinical deterioration and not by protocol as occurred in the group receiving antimalarials.

While severe cases of cytopenias were more frequent in the group that received antimalarial drugs, it cannot be solely attributed to these drugs. Regarding neuropsychiatric adverse effects, they have been observed in up to 12% of patients receiving antimalarials (Sato et al., 2020); however, we did not find any neuropsychiatric adverse effect in our study. This could be explained by underreported signs or symptoms in clinical records and by the limited interaction allowed with COVID-19 patients which compromised neurological examinations. In relation to hepatotoxicity in antimalarial users, few cases of liver failure have been described during the COVID-19 pandemic (Falcão et al., 2020). In our cohort, we did not find a difference in the change of the mean value per day for each liver function test between both groups. Finally, although the number of hypoglycemia cases were similar in boths groups, two cases of grade-4 hypoglycemia occurred among patients receiving antimalarials.

Previous studies have associated a higher in-hospital mortality in patients receiving antimalarials or macrolides for COVID-19. (Rosenberg et al., 2020). In this study almost all patients received macrolides and we found a similar mortality in both groups when restricting to patients who received optimal care (adjusted RR for mortality of 1.12, 95% CI 0.69—1.82. Although we performed an adjusted analysis that accounts for potential confounders, we acknowledge the existence of residual bias due to unmeasured confounders. However, our results are similar to those reported by Calvacanti; they did not find a difference in mortality, even when patients were also taking macrolides (Cavalcanti et al., 2020).

Nowadays, antimalarials are hardly recommended by any COVID-19 treatment guideline and it might seem obvious the evidence discouraging their use is compelling, nonetheless, real-life facts suggest the opposite. Recently Brazil have reported their use despite a lack of effectiveness (Kmietowicz, 2021). This study provides a real-life/pharmacovigilance experience with the use of these medications in the setting of a developing country.

## Limitations

We acknowledge the limitations of our study. Although significant outcomes (mortality, discharge, ICU requirement) were completely collected, most of the adverse effects data is incomplete. There was limited identification of adverse effects *via* electronic medical records, which may underestimate the true incidence, and there was scarcity of follow-up EKGs, which may underestimate the incidence of QTc interval prolongation. The reduction in the number of EKG assessments was considered to minimize healthcare workers’ exposure and because significant EKG abnormalities couldn't be solely accounted for the effect of antimalarial drugs. However, we consider it relevant to report these effects for future applications.

## Conclusion

The use of HCQ/CLQ during the first months of the COVID-19 pandemic was widespread, especially among mild to moderate cases. Although the adverse events in patients with COVID-19 treated with antimalarials were similar to those who did not receive antimalarials at our institution that has a rigorous pharmacological surveillance, they do not improve survival in patients who receive optimal medical care.

## Data Availability

The original contributions presented in the study are included in the article/Supplementary Material, further inquiries can be directed to the corresponding author.
