# Supplementary material for: Adverse Effects Associated With the Use of Antimalarials During The COVID-19 Pandemic in a Tertiary Care Center in Mexico City
Source: Front Pharmacol. 2021 Jun 3;12:668678. doi: 10.3389/fphar.2021.668678 (PMC8210417; doi:10.3389/fphar.2021.668678)
Supplement: Supplementary file 1 [file DataSheet1.docx]

**Supplementary Table S1. In-hospital additional treatment received by patients with COVID-19 treated with and without antimalarial drugs.**

| **Characteristic** | N | Overall, N = 626 | HCQ/CLQ, N = 235 | No antimalarial, N = 391 | p-value |
| --- | --- | --- | --- | --- | --- |
| **Macrolide** | 592 | 578 (98%) | 229 (97%) | 349 (98%) | >0.99 |
| **Carbapenem** | 536 | 4 (0.7%) | 1 (0.5%) | 3 (0.9%) | >0.99 |
| **β-lactam** | 546 | 540 (99%) | 211 (99%) | 329 (99%) | >0.99 |
| **Vancomycin** | 536 | 10 (1.9%) | 3 (1.4%) | 7 (2.2%) | 0.75 |
| **Oseltamivir** | 626 | 200 (32%) | 132 (56%) | 68 (17%) | **<0.001** |
| **Corticosteroids** | 626 | 38 (6.1%) | 11 (4.7%) | 27 (6.9%) | 0.34 |
| **Anticoagulation** | 626 |  |  |  |  |
| None |  | 36 (5.8%) | 14 (6.0%) | 22 (5.6%) | 0.88 |
| Prophylactic |  | 515 (82%) | 191 (81%) | 324 (83%) |  |
| Therapeutic |  | 75 (12%) | 30 (13%) | 45 (12%) |  |
| **Other (Clinical trials)** | 626 |  |  |  |  |
| Tocilizumab |  | 90 (14 %) | 34 (14%) | 56 (14%) |  |
| Colchicine |  | 9 (1.4%) | 5 (2%) | 4 (1%) |  |
| Remdesivir |  | 5 (0.7%) | 2 (0.8%) | 3 (0.7%) |  |
| Pirfenidone |  | 1 (1.5%) | 0 (0%) | 1 (0.2%) |  |
| Pyridostigmine |  | 12 (1.9%) | 0 (0%) | 12 (3%) |  |

HCQ, hydroxychloroquine; CLQ, chloroquine

**Supplementary Table S2. Electrocardiographic characteristics of patients with COVID-19 treated with and without antimalarial drugs in a tertiary care center in Mexico City.**

| **Baseline EKG** | N | Overall, N = 626 | HCQ/CLQ, N = 235 | No antimalarial, N = 391 | p-value |
| --- | --- | --- | --- | --- | --- |
| **QTc,** ms | 291 | 421 (37) | 422 (38) | 420 (36) | 0.73 |
| **Delta-QTc > 50 ms** | 132 | 16 (12%) | 16 (13%) | 0 (0%) | 0.38 |
| **HR,**  bpm | 292 | 85 (23) | 84 (15) | 87 (31) | 0.29 |
| **SVT** | 626 | 5 (0.8%) | 3 (1.3%) | 2 (0.5%) | 0.37 |
| **Ventricular arrhythmias** | 626 | 8 (1.3%) | 6 (2.6%) | 2 (0.5%) | 0.058 |

HCQ, hydroxychloroquine; CLQ, chloroquine; HR, heart rate; SVT, supraventricular tachycardia; QTc, QT corrected interval

**Supplementary Table 3. Clinical characteristics of patients with COVID-19 of patients with severe cardiac arrhythmias with and without antimalarial drugs in a tertiary care center in Mexico City.**

| Case | Age | Sex | BMI  kg/m^2^ | qSOFA | NIH  severity | Admission site | IMV | TnS  pg/mL | CPK  U/L | Therapy | Posology | LOS (days) | Macrolide | QT1 | QT2 | ΔQT > 50 ms | Arrhythmia | Outcome at discharge |
| --- | --- | --- | --- | --- | --- | --- | --- | --- | --- | --- | --- | --- | --- | --- | --- | --- | --- | --- |
| 1 | 54 | M | 25 | 1 | Severe | ICU | Yes | 6 | 319 | HCQ | 400 mg BID (1), 200 BID | 4 | Yes | - | - | - | PMTV | Death |
| 2 | 50 | F | 48 | 0 | Severe | Hospital ward | No | 2 | 57 | CLQ | 300 mg BID (1), 150 BID | 4 | Yes | 372 | 400 | No | MVE | Discharge |
| 3 | 57 | F | 29 | 1 | Severe | Hospital ward | No | - | - | CLQ | 300 mg BID (1), 150 BID | 1 | Yes | 400 | 475 | Yes | MVE | Discharge |
| 4 | 62 | M | 33 | 1 | Severe | ICU | Yes | 16 | 236 | CLQ | 300 mg BID (1), 150 BID | 5 | Yes | 448 | 483 | No | MVE | Discharge |
| 5 | 62 | M | 26 | 0 | Severe | Hospital ward | No | 10 | 665 | CLQ | 300 mg BID (1), 150 BID | 5 | Yes | 363 | 405 | No | PMTV | Death |
| 6 | 52 | M | 26 | 1 | Severe | Hospital ward | No | 3 | 41 | CLQ | 300 mg BID (1), 150 BID | 7 | Yes | 416 | - | - | VF | Death |
| 7 | 42 | M | 45 | 1 | Severe | ICU | Yes | 243 | 524 | - | - | - | - | - | - | - | MVE | Death |
| 8 | 45 | M | 33 | 1 | Severe | Hospital ward | Yes | - | - | - | - | - | - | - | - | - | PMTV | Death |

IMV, Invasive mechanical ventilation; TnS, High sensitivity Troponin; CPK creatine phosphokinase;LOS, length-of-stay; PMTV, Pulseless monomorphic ventricular tachycardia; VF ventricular fibrillation; MVE, Monomorphic ventricular extrasystole

**Supplementary Table S4. Safety outcomes of patients with COVID-19 treated with and without antimalarial drugs in a tertiary care center in Mexico City.**

| **Characteristics** | N | Overall, N = 626 | HCQ/CLQ, N = 235 | No antimalarial, N = 391 | p-value |
| --- | --- | --- | --- | --- | --- |
| **ICU requirement**- no. (%) | 626 | 269 (43%) | 88 (37%) | 181 (46%) | **0.037** |
| **IMV need -** no. (%) | 626 | 145 (23%) | 70 (30%) | 75 (19%) | **0.003** |
| **Shock** - no. (%) | 621 | 133 (21%) | 57 (24%) | 76 (20%) | 0.20 |
| **RRT need** - no. (%) | 622 | 24 (3.9%) | 6 (2.6%) | 18 (4.7%) | 0.27 |
| **LOS (days)** - no. (%) | 626 | 7 (4, 13) | 9 (5, 14) | 6 (3, 12) | **<0.001** |

HCQ, hydroxychloroquine; CLQ, chloroquine; ICU, intensive care unit; IMV, invasive mechanical ventilation; RRT, renal replacement therapy; AE, adverse effects; LOS, length-of-stay.
